# NAD(P)H quinone oxidoreductase (NQO1): an enzyme which needs just enough mobility, in just the right places

**DOI:** 10.1042/BSR20180459

**Published:** 2019-01-03

**Authors:** Angel L. Pey, Clare F. Megarity, David J. Timson

**Affiliations:** 1 Department of Physical Chemistry, Faculty of Sciences, University of Granada, Av. Fuentenueva s/n, Granada 18071, Spain; 2 Medical Biology Centre, School of Biological Sciences, Queen’s University Belfast, 97 Lisburn Road, Belfast BT9 7BL, U.K.; 3 School of Pharmacy and Biomolecular Sciences, University of Brighton, Huxley Building, Lewes Road, Brighton BN2 4GJ, U.K.

**Keywords:** cancer-associated mutation, DT-diaphorase, negative cooperativity, protein mobility, quinone oxidoreductase

## Abstract

NAD(P)H quinone oxidoreductase 1 (NQO1) catalyses the two electron reduction of quinones and a wide range of other organic compounds. Its physiological role is believed to be partly the reduction of free radical load in cells and the detoxification of xenobiotics. It also has non-enzymatic functions stabilising a number of cellular regulators including p53. Functionally, NQO1 is a homodimer with two active sites formed from residues from both polypeptide chains. Catalysis proceeds via a substituted enzyme mechanism involving a tightly bound FAD cofactor. Dicoumarol and some structurally related compounds act as competitive inhibitors of NQO1. There is some evidence for negative cooperativity in quinine oxidoreductases which is most likely to be mediated at least in part by alterations to the mobility of the protein. Human NQO1 is implicated in cancer. It is often over-expressed in cancer cells and as such is considered as a possible drug target. Interestingly, a common polymorphic form of human NQO1, p.P187S, is associated with an increased risk of several forms of cancer. This variant has much lower activity than the wild-type, primarily due to its substantially reduced affinity for FAD which results from lower stability. This lower stability results from inappropriate mobility of key parts of the protein. Thus, NQO1 relies on correct mobility for normal function, but inappropriate mobility results in dysfunction and may cause disease.

## Introduction: NQO1

**Figure 1:**
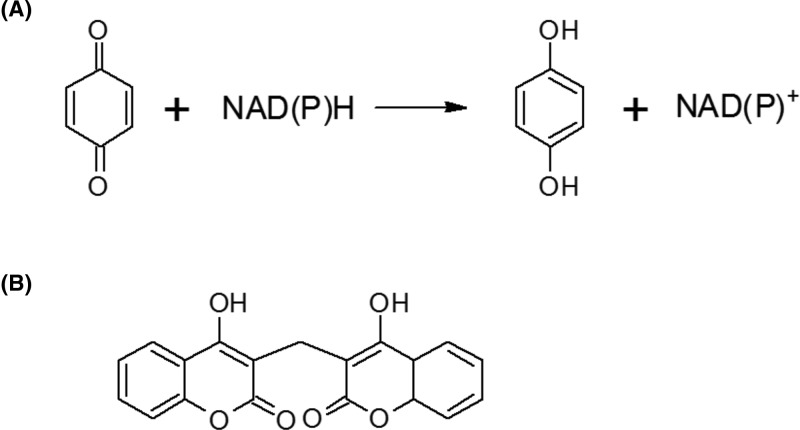
The reaction catalysed by NQO1 (**A**) The general reaction catalysed by quinone oxidoreductases in which a quinone is reduced to a hydroquinone by the NADH or NADPH. Human NQO1 is able to catalyse a great variety and diversity of substrates. (**B**) The structure of the potent inhibitor of NQO1, dicoumarol. This compound has been widely used in experimental studies of NQO1 inhibition. The figure shows one of the various possible tautomeric and ionic forms of the molecule [45,87,88].

NAD(P)H quinone oxidoreductase (NQO1; DT-diaphorase; EC 1.6.5.2) is an intracellular, cytosolic enzyme which catalyses the reduction of quinones and a wide variety of other compounds (Figure 1A) [1]. In general these are two electron reductions which, in the case of quinones, avoid the production of reactive semiquinones [2,3]. Unusually, the enzyme works with almost equal efficiency with the cofactors NADH and NADPH [4]. Catalytically the enzyme’s function requires a tightly bound FAD cofactor. This is reduced by NAD(P)H in the first stage of a substituted enzyme (ping-pong) mechanism. The oxidised cofactor (NAD(P)^+^) then leaves the active site enabling the second substrate to enter. This second substrate is then reduced by the FADH_2_ [5]. In addition to quinones, NQO1 can catalyse the reduction of nitroaromatic compounds, imidazoles and iron (III) ions [6–8]. In humans there is a second enzyme, ribosyldihydronicotinamide quinone oxidoreductase 2 (NQO2; EC 1.10.5.1) which shares considerable sequence and structural similarity to NQO1 [9]. However, this enzyme does not function with NAD(P)H as a cofactor, but uses ribosyldihydronicotinamide (reduced nicotinamide ribonucleotide; NRH) in its place [10]. Bacteria often have several enzymes with quinone oxidoreductase activity [10]. The ‘nitroreductase’ group of enzymes fall into his class, although they are named for their ability to catalyse the reduction of some nitrogenous organic compounds [11]. They have attracted interest due to their potential to activate anti-cancer prodrugs [12]. The modulator of drug activity (Mda) proteins have greater structural similarity to mammalian NQO1 and also have quinone oxidoreductase activity [13–16]. Despite the broad substrate ranges of these various quinone oxidoreductases they have, so far, attracted relatively little interest as possible biocatalysts. Their ability to catalyse the reduction of a wide range of organic compounds (and some inorganic ones) without modification to the enzyme, suggests that they have considerable potential in this area.

**Figure 2:**
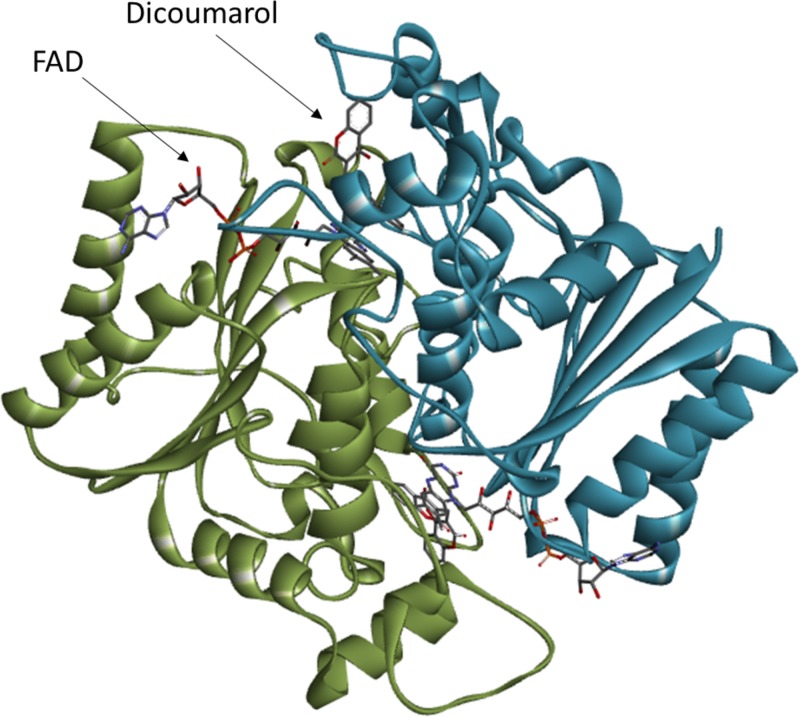
The overall structure of human NQO1 The figure shows the dimeric form of NQO1 bound to dicoumarol (PDB: 2F1O [18]). The two subunits of the homodimer are shown in blue and green. The FAD cofactor and dicoumarol inhibitor are shown in one active site. The second active site lies on the opposite side of the protein. The image was created using Discovery Studio Visualiser 4.5 (Dassault Systèmes BIOVIA).

Structurally, NQO1 is a homodimer with two active sites each located at the interface between the subunits (Figure 2) [17,18]. Thus, both active sites comprise residues from both subunits. The FAD cofactor forms part of these active sites and the NAD(P)H substrate binds in such a way that the nicotinamide ring lies parallel to the FAD, facilitating efficient electron transfer [17]. The anticoagulant dicoumarol is a potent inhibitor of NQO1 (*K*_i_ = 50 pM as determined by inhibition studies on the rat enzyme; *K*_d_ = 120 nM as determined by isothermal titration calorimetry on the human enzyme) [5,19]. This compound also binds in a conformation which partially overlaps the FAD cofactor (Figure 1B). This provides a structural explanation for this compound’s ability to act as a competitive inhibitor of the enzyme with respect to NAD(P)H [1,18].

The *in vivo* roles of NQO1 have probably not been fully elucidated. It is known to play a minor role in the blood clotting cycle, reducing vitamin K to vitamin K hydroquinone [20]. However, the majority of this transformation is catalysed by vitamin K oxidoreductase (VKOR; EC1.17.4.4) [21]. NQO1 is often up-regulated in response to cellular stress and it is a reasonable hypothesis that it has a role in minimising free radical load within cells [22,23]. It is also likely to play a role in the detoxification of xenobiotics [24–28].

Reduced NQO1 activity is associated with a predisposition to cancer. In particular, a polymorphism which results in the change of a proline to a serine residue at position 187 (p.P187S) has been associated with many different types of cancers in a large number of studies across several population groups [29]. According to the Ensembl database, the polymorphism occurs in approximately 25% of the global human population and is particularly common in people of Chinese ethnicity (approximately 50%) [30]. Interestingly, NQO1 activity is increased in some forms of cancer [31]. This may be linked to the increased free radical load in rapidly metabolising cancer cells. Thus inhibition of NQO1 by dicoumarol and other compounds has, perhaps paradoxically, been demonstrated to kill pancreatic cancer cells [32–36]. Considerable efforts have been made to identify other molecules which act as selective inhibitors of NQO1 (for examples, see [34–44]). (Dicoumarol is also an effective inhibitor of VKOR and acts as a mitochondrial uncoupling agent – hence the need for compounds which demonstrate greater specificity towards NQO1 [45–48].) Some anti-cancer drugs (e.g. mitomycin C and 3-hydroxy-5-aziridinyl-1-methyl-2 (1*H*-indole-4,7-dione)prop-β-en-α-ol or EO9) are reductively activated by NQO1, a fact which means that their activity will be higher in cancer cells which over-express the enzyme [49–52].

NQO1 also has non-enzymatic roles. It stabilises the cell cycle regulator and tumour suppressor protein p53, increasing its cellular half-life [53–55]. This interaction is antagonised by dicoumarol [54]. Thus, less stable and active forms of NQO1 (e.g. the p.P187S polymorphic form), are likely to be less effective at stabilising p53, providing another explanation for the increased cancer risks associated with this mutation. In addition to p53, NQO1 is also known to interact with, and stabilise, another tumour suppressor protein p73 and the enzyme ornithine decarboxylase (EC 4.1.1.17) which catalyses the first committed step in polyamine biosynthesis [56,57]. NQO1 interacts with, and regulates, proteosomal components suggesting a role in controlling the degradation of proteins in the cell [58].

The structure, function, role in disease and drug interactions of NQO1 have been extensively reviewed (e.g. [59–66]). Here, we focus on a less-explored aspect of the enzyme – its mobility and how this affects its functions and plays a part in the enzyme’s roles in cancer pathology.

### NQO1 mobility in the right places

It is becoming increasingly accepted that the mobility of proteins is just as important as their structures in facilitating their functions [67]. For example, backbone flexibility and the motions of side chains in the active site is often critical in catalysis [67, 68]. In some cases, domains move relative to one another to facilitate binding or catalysis. Furthermore, while information can be transmitted within proteins (and protein complexes) by conformational changes, it is also the case that this can be achieved by alterations to mobility which propagate through the structure [67].

There is some evidence that NQO1 is negatively cooperative towards inhibitors such as dicoumarol. The rat enzyme demonstrates non-linear Scatchard plots in binding assays and non-linear Lineweaver–Burke plots in enzyme assays [69]. Negative cooperativity has also been observed between human NQO1 and the FAD cofactor [70]. Thus, it is tempting to speculate that negative cooperativity towards both cofactor and inhibitors (which bind adjacent to that cofactor [18]) arise from the same, or related, causes. Negative cooperativity towards inhibitors has also been observed in human NQO2 and the *Saccharomyces cerevisiae* nitrogenase-like enzyme Lot6p [71,72].

The biological significance of this negative cooperativity is not yet known. In general, negative cooperativity functions to ‘dampen’ the response of a system to changes in concentration of the effector molecule [73,74]. To date, no naturally occurring cellular inhibitors of NQO1 have been discovered. Thus, there is the possibility that the negative cooperativity observed with compounds like dicoumarol is an artefact, perhaps resulting from the cooperativity in FAD binding. Alternatively, it may play a key role in the regulation of this enzyme by as yet unidentified small molecule inhibitors in the cell. There is increasing evidence that NQO1 plays a role in sensing and responding to the cell’s redox state [75]. Thus, this negative cooperativity with FAD may be important in the sensing of the cell’s energetic status and overall FAD content.

Structurally, negative cooperativity requires communication between the enzyme’s active sites. The crystal structure of NQO1 in complex with dicoumarol shows both active sites bound to the inhibitor [18]. Presumably this occurs due to the relatively high concentrations of dicoumarol used in the production of the crystals. However, this structure provides no clues about any conformational changes which may enable information exchange between the active sites. This would require a structure with only one site occupied per NQO1 homodimer. In the yeast quinone oxidoreductase Lot6p, an α-helix lies close to the resveratrol binding site. Alteration of a glycine residue in this helix to serine reduces the negative cooperativity of the enzyme [72]. Since serine residues generally impart lower conformational flexibility than glycine, it seems likely that communication in Lot6p is mediated in part by alterations in protein mobility [76]. It is tempting to speculate that similar mechanisms may occur in mammalian NQO1.

### NQO1 mobility in the wrong places

Early work on the p.P187S variant of human NQO1 (also known as the NQO1*2 variant) revealed substantially reduced activity and cellular concentrations of the enzyme [77,78]. This strongly suggested that this amino acid change results in reduced protein stability and thus increased cellular degradation of the enzyme. This hypothesis has been subsequently supported through biochemical and biophysical studies. The thermal stability as estimated by the ‘melting temperature’ (determined by differential scanning calorimetry, differential scanning fluorimetry and far-UV circular dichroism spectroscopy) is approximately 8 K lower in p.P187S compared with the wild-type [79]. This variant is also much more susceptible to limited proteolysis than the wild-type [19,79]. Interestingly, the crystal structure of the p.P187S variant (PDB: 4CF6) shows relatively few changes compared with the wild-type [80]. However, NMR studies suggest that the structure is considerably more mobile and prone to unfolding [80]. Biochemical studies have shown that the FAD content of p.P187S is substantially reduced compared with the wild-type [79]. The affinity for this cofactor is reduced approximately 10- to 50-fold [70,81,82].

**Figure 3:**
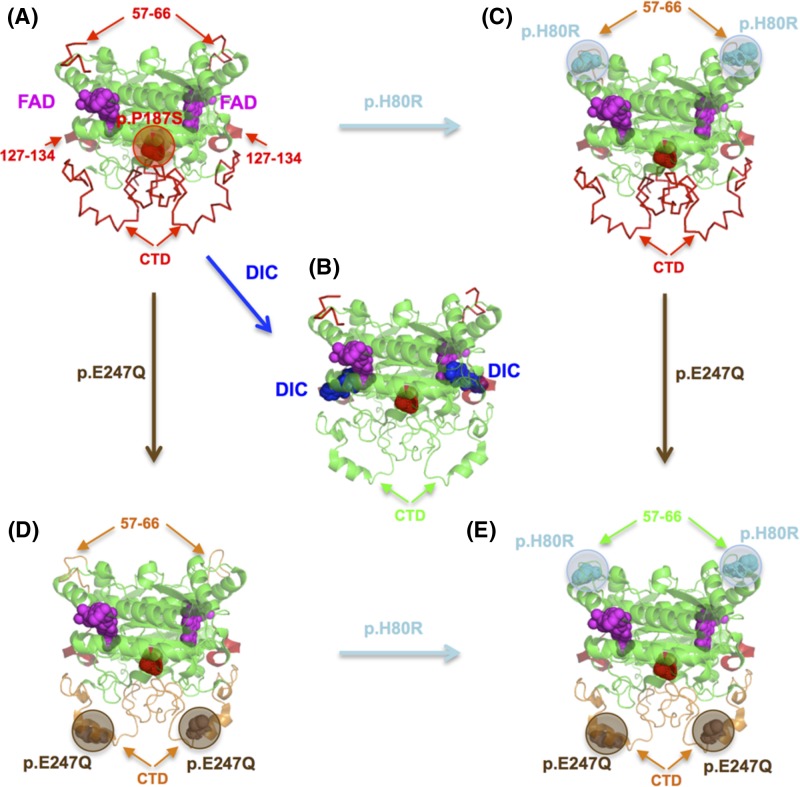
Alterations in conformation and dynamics due to p.P187S and its correction by dicoumarol binding and the suppressor mutations p.H80R and p.E247Q (**A**) p.P187S primarily affects three structural regions (highlighted in red with arrows): (i) the dynamics of the loop 57–66 in the apo-state; (ii) the dynamics of the region 127–134 in the holo-state; (iii) the C-terminal domain (CTD) is partially unfolded and highly dynamic in the holo-state; (**B**) dicoumarol binding induces the folding of the CTD in the holo-state (highlighted in green); (**C**) the suppressor mutation p.H80R partially corrects FAD binding affinity by dynamic stabilisation of the loop 57–66 in the apo-state (in orange); (**D**) the suppressor mutation p.E247Q stabilises the CTD in the holo-state and dynamically stabilises the loop 57–66 in the apo-state (highlighted in orange); (**E**) the suppressor mutations in *cis* lead to additive correction of both FAD binding and CTD stability. DIC, dicoumarol.

Pro-187 is located in a buried region close to the monomer:monomer interface (Figure 3A). Any alteration of the conformationally restrained proline will be expected to lead to increased mobility, in addition to alterations in local protein interactions due to perturbed protein packing. Indeed, these structural and energetic perturbations at Pro187 are sensed in distal sites affecting their conformation and dynamics (Figure 3A) [19]. Structural and dynamic and perturbation of the monomer:monomer interface due to p.P187S decreases its conformational stability [19], while more damaging unnatural mutations such as p.P187R, p.P187E and p.P187L prevent stable folding into dimers [83]. In addition, these perturbations due to p.P187S affect two functional regions: those affecting the dynamics of the FAD binding site in the N-terminal domain (particularly dynamic alterations at the loop comprising residues 57–66 and the region 127–134) and those decreasing the conformational stability of the C-terminal domain leading to its partial unfolding (Figure 3A) [19,80,84]. An intriguing, but not yet explored, possibility is that the region 127–134 acts as a link for communication of p.P187S mediated perturbations of the FAD binding site and the C-terminal domain (Figure 3A). The dynamic perturbations at the N-terminal domain affect regions close to the FAD binding site, particularly the loop 57–66, increasing its dynamics in the apo-state (Figure 3A). Consequently, to bind FAD, p.P187S is required to *constrain* the conformation of the FAD binding site to a larger extent, leading to an entropic penalty to binding [19, 81]. The effects of p.P187S on the conformational stability and dynamics of the C-terminal domain are associated with enhanced polyubiquitylation and the proteasomal degradation of the polymorphic variant (Figure 3A) [19,78,80,85]. Binding of dicoumarol to the polymorphic variant has structural and energetic signatures consistent with folding of the C-terminal domain upon inhibitor binding, which protected p.P187S towards degradation through the ubiquitin-dependent proteasomal degradation (Figure 3B) [19,84,85].

Local stabilisation of the 57–66 loop by the evolutionary divergent and suppressor mutation p.H80R leads to partial rescue of the FAD binding affinity of p.P187S (Figure 3C) through a population shift in the conformational ensemble of the apo-state towards binding competent states involving changes in protein dynamics [81,82]. In addition, a second suppressor mutation located at the C-terminal domain alone (p.E247Q) has been shown to partially restore the folding state of the C-terminal domain and reduced its dynamics (Figure 3D) [82], which would protect the polymorphic variant from degradation (ALP lab, work in progress). Interestingly, the stabilisation of the C-terminal domain due to p.E247Q is also communicated to the distal FAD binding site (located 25 Å from residue 247) further supporting long-range communication of conformational and dynamic information between distal functional sites in NQO1. Consistently, when the C-terminal domain was deleted by mutagenesis, the effects of p.P187S on FAD binding site located at the N-terminal domain were abolished, suggesting the existence of an allosteric network in the NQO1 protein which is perturbed by the polymorphic variant [84]. Plausibly, the communication of the local stabilising effect of p.E247Q to the FAD binding site occurs through this network, cooperating with p.H80R in rescuing FAD binding in the polymorphic variant (both suppressor mutations in *cis* increase FAD binding affinity by 20-fold in p.P187S (Figure 3E) [70,84]. Interestingly, most of the effects of p.P187S and the suppressor mutations seem to operate through the apo-state conformational ensemble, which is characterised by low conformational stability and high conformational dynamics, suggesting that most of these mutational effects have dynamic as well as structural basis [19,81,82,84]. It is also worth noting that rodent NQO1 contains by default the suppressor amino acids Gln-247 and Arg-80, which suggested that some mammalian NQO1 orthologues (containing these consensus amino acids) would be more robust that the human enzyme towards p.P187S through the presence of these evolutionary divergent gatekeeper amino acids in conformational and dynamic terms [82,86].

## Conclusions

Like most enzymes, the flexibility of NQO1 is critical to its function. Mobility is required for catalysis and is likely to mediate communication between the active sites. However inappropriate flexibility can result in dysfunction – failure to fold and the consequent dramatically reduced affinity for FAD. This increases the risk of various types of cancer. A more complete understanding of the interplay between mobility, catalysis, inhibition and NQO1’s non-enzymatic functions is critical to understand this protein’s role in health and disease. This includes the potential to address the dysfunction of the p.P187S variant using small molecules designed to stabilise the protein (pharmacological chaperones) and the design of drugs to inhibit NQO1 specifically in cancer patients. It may also be important in any future biotechnological applications, such as biocatalysis. In these applications it may be necessary to stabilise the protein to increase its lifetime in industrial processes and also modulate the specificity of the active sites.

## Abbreviations

NQO1: NAD(P)H quinone oxidoreductase 1
VKOR: vitamin K oxidoreductase
